# Time-saving opportunities in knee osteoarthritis: T_2_ mapping and structural imaging of the knee using a single 5-min MRI scan

**DOI:** 10.1007/s00330-019-06542-9

**Published:** 2019-12-16

**Authors:** Susanne M. Eijgenraam, Akshay S. Chaudhari, Max Reijman, Sita M. A. Bierma-Zeinstra, Brian A. Hargreaves, Jos Runhaar, Frank W. J. Heijboer, Garry E. Gold, Edwin H. G. Oei

**Affiliations:** 1 Deptartment of Radiology & Nuclear Medicine, Erasmus MC, University Medical Center, Dr. Molewaterplein 40, Room Nd-547, 3015 GD Rotterdam, The Netherlands; 2 Deptartment of Orthopedic Surgery, Erasmus MC, University Medical Center, Rotterdam, The Netherlands; 3 Deptartment of Radiology, Stanford University, Stanford, CA USA; 4 Deptartment of General Practice, Erasmus MC, University Medical Center, Rotterdam, The Netherlands; 5 Deptartment of Electrical Engineering, Stanford University, Stanford, CA USA; 6 Deptartment of Bioengineering, Stanford University, Stanford, CA USA; 7 Deptartment of Orthopedic Surgery, Stanford University, Stanford, CA USA

**Keywords:** Knee, Cartilage, Meniscus, Osteoarthritis, Magnetic resonance imaging

## Abstract

**Objectives:**

To assess the discriminative power of a 5-min quantitative double-echo steady-state (qDESS) sequence for simultaneous T_2_ measurements of cartilage and meniscus, and structural knee osteoarthritis (OA) assessment, in a clinical OA population, using radiographic knee OA as reference standard.

**Methods:**

Fifty-three subjects were included and divided over three groups based on radiographic and clinical knee OA: 20 subjects with no OA (Kellgren-Lawrence grade (KLG) 0), 18 with mild OA (KLG2), and 15 with moderate OA (KLG3). All patients underwent a 5-min qDESS scan. We measured T_2_ relaxation times in four cartilage and four meniscus regions of interest (ROIs) and performed structural OA evaluation with the MRI Osteoarthritis Knee Score (MOAKS) using qDESS with multiplanar reformatting. Between-group differences in T_2_ values and MOAKS were calculated using ANOVA. Correlations of the reference standard (i.e., radiographic knee OA) with T_2_ and MOAKS were assessed with correlation analyses for ordinal variables.

**Results:**

In cartilage, mean T_2_ values were 36.1 ± SD 4.3, 40.6 ± 5.9, and 47.1 ± 4.3 ms for no, mild, and moderate OA, respectively (*p* < 0.001). In menisci, mean T_2_ values were 15 ± 3.6, 17.5 ± 3.8, and 20.6 ± 4.7 ms for no, mild, and moderate OA, respectively (*p* < 0.001). Statistically significant correlations were found between radiographic OA and T_2_ and between radiographic OA and MOAKS in all ROIs (*p* < 0.05).

**Conclusion:**

Quantitative T_2_ and structural assessment of cartilage and meniscus, using a single 5-min qDESS scan, can distinguish between different grades of radiographic OA, demonstrating the potential of qDESS as an efficient tool for OA imaging.

**Key Points:**

*• Quantitative T*_*2*_*values of cartilage and meniscus as well as structural assessment of the knee with a single 5-min quantitative double-echo steady-state (qDESS) scan can distinguish between different grades of knee osteoarthritis (OA).*

*• Quantitative and structural qDESS-based measurements correlate significantly with the reference standard, radiographic degree of OA, for all cartilage and meniscus regions.*

*• By providing quantitative measurements and diagnostic image quality in one rapid MRI scan, qDESS has great potential for application in large-scale clinical trials in knee OA.*

**Electronic supplementary material:**

The online version of this article (10.1007/s00330-019-06542-9) contains supplementary material, which is available to authorized users.

## Introduction

The growing population suffering from knee osteoarthritis (OA) and the lack of early biomarkers and therapeutics prompt the need for efficient imaging methods [1]. Magnetic resonance imaging (MRI) allows assessment of the whole knee joint, making it ideally suited for imaging in knee OA, which is a multi-tissue disease [2, 3]. Several potential MRI-based biomarkers have been proposed in this context [4]. In particular, the role of quantitative MRI (qMRI) techniques is emerging. qMRI techniques, such as T_2_ mapping, have the ability to non-invasively detect subtle changes in biochemical composition of tissues such as cartilage and menisci. Increased T_2_ relaxation times have been shown to be associated with cartilage and meniscus degeneration, potentially enabling early-stage detection of knee OA and similar conditions [5–8]. T_2_ mapping does not require a contrast injection or special MRI imaging hardware and numerous techniques for post-processing of T_2_ images are available [5, 7, 9, 10].

Besides quantitative MR imaging, structural evaluation of the knee is fundamental in the assessment of knee OA, given its multi-tissue nature [2, 3]. The semi-quantitative MRI Osteoarthritis Knee Score (MOAKS) [11] is a widely used and well-validated instrument for evaluating knee OA and has been applied in large-scale epidemiological OA studies such as the Osteoarthritis Initiative (OAI) [11–14].

T_2_ mapping and MOAKS are potential biomarkers to non-invasively assess joint health; however, acquiring them efficiently is a challenge. In general, multiple sequences are used in knee OA imaging, often resulting in time-consuming MRI protocols that take 30–45 min or longer [6, 15]. In particular, in the context of large-scale clinical trials and repeated measurements, MRI acquisition can create a significant burden for patients, hospitals, and research budgets. In the context of quantitative MRI, multiple sequences also bring up the need for registration between sequences. Hence, creating more streamlined MRI protocols and reducing acquisition time are of great interest.

In the present study, we evaluated a novel MRI technique to reduce scan time in the context of knee OA: the quantitative double-echo steady-state (qDESS) sequence. qDESS generates two echoes: one echo with T_1_/T_2_ weighting (resembling proton-density contrast) and one echo with T_2_ weighting. It has the potential to provide diagnostic images as well as quantitative measurements (i.e., T_2_ maps) of the knee in a single sequence with an acquisition time less than 5 min [16, 17].

Proof-of-concept of qDESS for T_2_ mapping of cartilage and meniscus and structural knee assessment (using MOAKS) has been provided by Chaudhari et al [16]. Focusing on healthy subjects, they validated qDESS against routine methods for T_2_ measurements and MOAKS and reported high accuracy in most tissues. Also, a pilot study in 10 patients with knee OA, performed in the same work, provided promising qDESS-based T_2_ mapping and MOAKS outcomes, suggesting that accurate knee OA measurements are possible with qDESS [16]. Building upon this work, we further assessed the discriminative power of quantitative and structural qDESS-based biomarkers, in a larger OA cohort against radiography, widely accepted as the gold standard for knee OA imaging [18, 19]. We evaluated structural MOAKS scores and T_2_ measurements of the knee cartilage and meniscus in a clinical OA population. In contrast to the approach of Chaudhari and colleagues, which comprised a global assessment of cartilage and menisci, in the present study, we evaluated predefined subregions of cartilage and menisci. Regional assessment is relevant as knee OA is a focal disease with a heterogenous disease pattern [6, 20, 21].

The aim of the present study was to assess the discriminative power of a single 5-min qDESS MRI sequence for simultaneous T_2_ measurements of cartilage and meniscus, and structural knee OA assessment, in a clinical OA population, using radiographic knee OA as reference standard.

## Methods

### Study population

This study was performed with approval from our Institutional Review Board and in compliance with Health Insurance Portability and Accountability Act (HIPAA) regulations. Written informed consent was obtained from all participants after receiving full explanation about the study. Consecutive patients who were referred by the Department of Orthopedic Surgery for knee MRI at Stanford Medical Center between December 2016 and July 2017 were screened for eligibility. The eligibility criteria for this study are shown in Table 1. Based on radiographic (Kellgren and Lawrence grade (KLG) [22]) and clinical (American College of Rheumatology (ACR) criteria [23]) degree of knee OA, three subject groups were selected: subjects with no OA (KLG0 and ACR negative), subjects with mild OA (KLG2 and ACR positive), and subjects with moderate OA (KLG3 and ACR positive).

**Table 1:** Eligibility criteria

Non-OA subjects	OA subjects
Referred for knee MRI	Referred for knee MRI
No contra-indication for MRI	No contra-indication for MRI
AP weight-bearing radiograph of index knee^a^ available	AP weight-bearing radiograph of index knee^a^ available
No ACL reconstruction in index knee in medical history	No ACL reconstruction in index knee in medical history
KLG0	KLG2 or KLG3
	Knee pain + at least 1 out of 3 following criteria:
	1. Age > 50 years
	2. Stiffness < 30 min
	3. Crepitus

^a^Acquired within 2 weeks before or after MRI acquisition

*OA*, osteoarthritis; *MRI*, magnetic resonance imaging; *AP*, anteroposterior; *ACL*, anterior cruciate ligament; *KLG*, Kellgren Lawrence grade

### Scoring of radiographic knee OA

The assessment of radiographic knee OA was performed according to the KLG criteria [22], by a researcher with a medical degree and 4 years of experience in musculoskeletal imaging research (SE) who was blinded to any patient data. Standardized, weight-bearing AP radiographs were used. A second reader, a musculoskeletal radiologist with 15 years of experience (EO), also performed the KL grading in a random selection of 20 subjects from the study population to assess inter-observer reliability. To assess intra-observer reliability of the primary observer (SE), 20 randomly selected subjects from the study population were re-evaluated 14 days after initial grading.

### MRI data acquisition

**Fig. 1:**
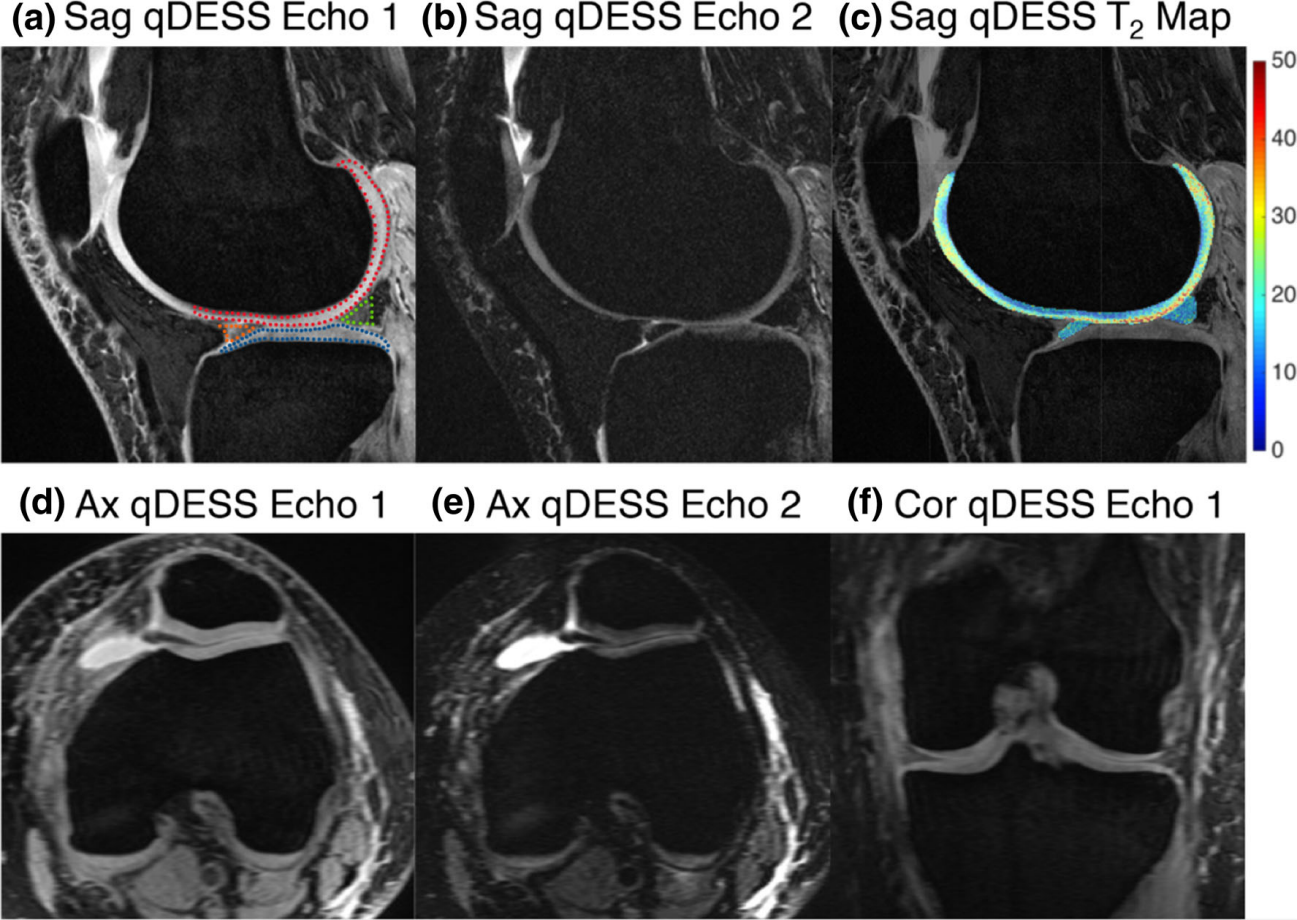
Representative example of first (**a**) and second (**b**) sagittal qDESS echo in a 37-year-old female without OA, lateral compartment. In **a**, femoral cartilage ROI is indicated by red dots, tibial cartilage ROI is indicated by blue dots, anterior meniscal horn is indicated by orange dots, and posterior meniscal horn is indicated by green dots. **c** Corresponding T_2_ colormaps of femoral cartilage and the anterior and posterior horns of the lateral meniscus (color bar on the right shows the range of T_2_ values). Sagittal qDESS images are used to generate reformatted reconstructions in the axial (**d**, **e**) and coronal (**f**) plane. Sag = sagittal; Ax = axial; Cor = coronal

MR imaging was performed on one of two identical 3-Tesla MR scanners (Discovery MR750, GE Healthcare), using a 5-min 3D sagittal qDESS scan with an 8-channel transmit-receive knee coil (InVivo). qDESS generates two echoes per repetition time: S+ (with T_1_/T_2_ contrast; echo time (TE) 5.7 ms; Fig. 1a) and S− (with T_2_ weighting; TE 30.1 ms; Fig. 1b) [16]. The sagittal qDESS images were used to generate axial and coronal reformats (Fig. 1d–f). Sequence parameters of qDESS are described in Table 2.

**Table 2:** qDESS MRI sequence parameters

Sequence parameter	qDESS
Matrix (RO × PE)	416 × 512
IN-plane resolution (mm^2^)	0.42 × 0.31
Slice thickness (mm)	1.5
Number of slices	80
TE S+, TE S− (ms)	5.7, 30.1
Number of echoes	2
TR (ms)	17.9
Flip angle (°)	20
Bandwidth (± kHz)	42
Parallel imaging	2 × 1
% corners cut	25
Scan time (mm:ss)	04:48

*qDESS*, quantitative double-echo steady-state; *MRI*, magnetic resonance imaging; *RO*, readout; *PE*, phase encodes; *TE*, echo time; *TR*, repetition time

### Quantitative MRI analysis (T_2_ mapping)

The two echoes of qDESS were used to compute T_2_ relaxation time parameter maps, by inverting the qDESS signal model [24]. qDESS T_2_ measurements have shown to have high concordance with multi-echo spin echo T_2_ measurements [25] and limited sensitivity to T_1_ and signal-to-noise ratio variations in cartilage and meniscus [26]. The first echo (S+) of sagittal qDESS was used for manual segmentation of cartilage and menisci for the calculation of T_2_ relaxation times (Fig. 1c). Segmentation was performed on single slices, by the same researcher (SE) blinded for the patient’s clinical data. For femoral and tibial cartilage segmentation, the centermost slice through the medial and lateral femoral condyle (defined as the slice midway between the slice on which the femoral condyle was first visible and the slice on which the femoral condyle was last visible) was identified. Four cartilage regions of interest (ROIs) were defined per patient: medial and lateral femoral cartilage and medial and lateral tibial cartilage. Trochlear cartilage was not included in quantitative analysis because of the potential influence of the magic angle effect on T_2_ relaxation times [27].

For meniscus segmentation, the sagittal slice depicting the maximum dimension of the anterior horn and posterior horn as individual triangles was used. Four meniscus ROIs were defined per patient: the anterior and posterior horn of the medial and lateral menisci. To avoid partial volume effects of joint fluid in case of a meniscal tear, the torn area was not included in segmentation. All segmentations and subsequent T_2_ analyses were performed using custom in-house software created in MATLAB (version R2011b; The Math-Works).

### Structural analysis of knee OA (MOAKS)

Structural, semi-quantitative assessment of cartilage and meniscus was performed using MOAKS [11] by the same researcher (SE). Both qDESS echoes with multiplanar reformatting were used. Criteria for MOAKS grading for cartilage (MOAKS_cartilage_) and meniscus (MOAKS_meniscus_), used in this study, are described in Supplementary Materials 1 and 2, respectively. We performed no second reading because high intra- and inter-observer reproducibility for MOAKS scoring using qDESS with separated echoes, especially for cartilage and meniscus, was reported in a previous study [16].

### Statistical analysis

We assessed the intra- and inter-observer reproducibility for KLG scoring by calculating weighted Cohen’s kappa. Tests for normality of baseline characteristics and outcomes were performed using Shapiro-Wilk tests. Between-group differences in overall (i.e., pooled across all ROIs) T_2_ values and MOAKS scores were evaluated using ANOVA (for parametric data) or Kruskal-Wallis tests (for non-parametric data). In case of statistically significant differences in mean age and/or sex among the three subject groups, a multivariate model with linear regression was used to assess the potential influence of these differences on T_2_ values and MOAKS scores. Associations between radiographic OA and T_2_ values and between radiographic OA and MOAKS were assessed in predefined cartilage and meniscus ROIs, and for overall scores using correlation analysis for ordinal variables (Spearman’s correlation). Differences were considered statistically significant at *p* < 0.05. All statistical analyses were performed using SPSS (version 24.0.0.0, 2018).

## Results

### Characteristics of study population

**Fig. 2:**
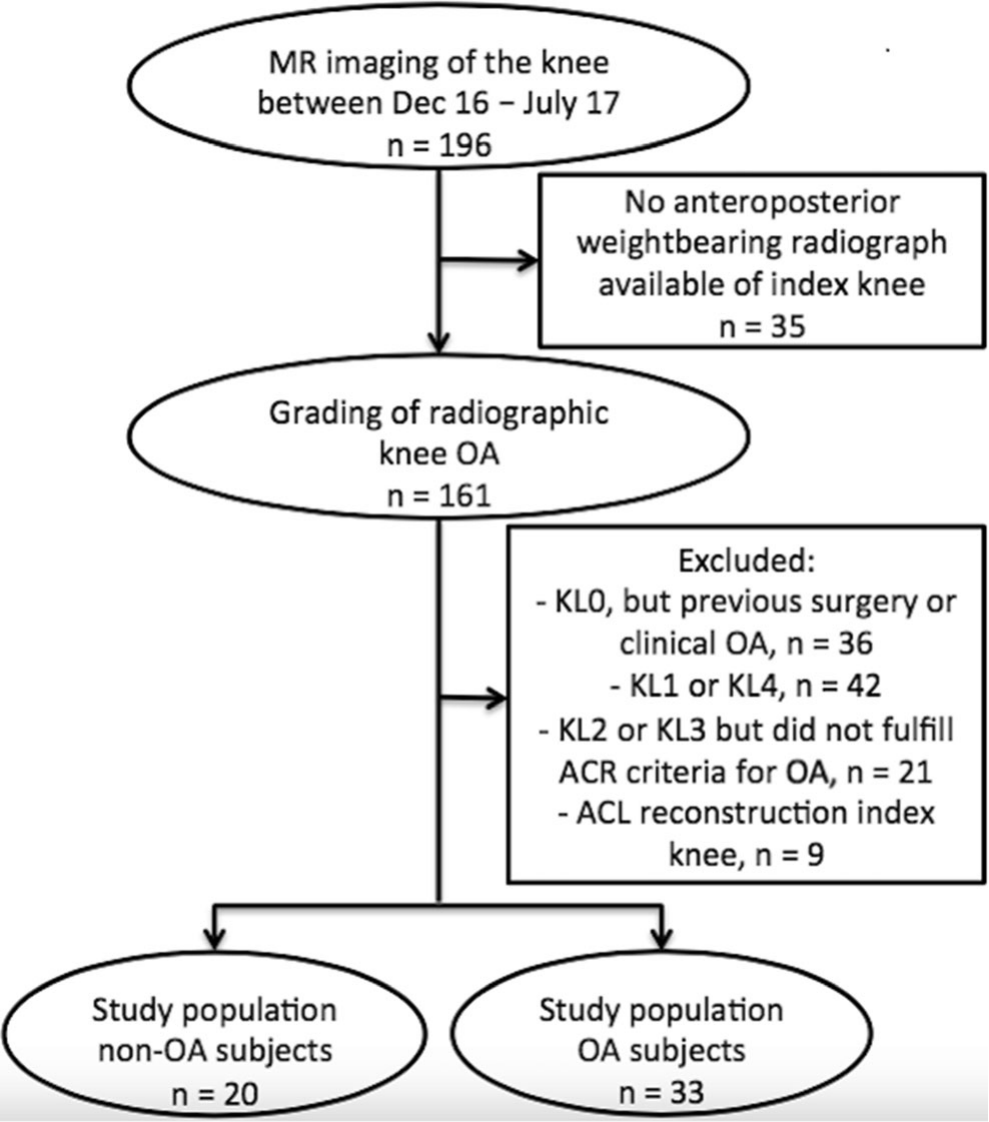
Flowchart showing the selection process of the study population. In the rectangles on the right, the number and nature of exclusions are described. MR = magnetic resonance; Dec = December; OA = osteoarthritis; KL = Kellgren and Lawrence grade; ACR = American College of Rheumatology; ACL = anterior cruciate ligament

Out of the 196 potentially eligible patients, 53 subjects were included in this study: 20 subjects with no knee OA, 18 subjects with mild knee OA, and 15 subjects with moderate knee OA. A flowchart of the selection of the study population is presented in Fig. 2. Characteristics of study participants, stratified by degree of OA, are summarized in Table 3. There was a slight overall male predominance of 60%, yet no statistically significant differences in sex distribution were found across the three subject groups. The mean age of patients with mild and moderate OA was statistically significantly higher (*p* < 0.001) compared with subjects with no OA. No statistically significant association between age and T_2_ values or MOAKS scores was found (data not shown).

**Table 3:** Characteristics of the study population, stratified by degree of OA

	No knee OA	Mild knee OA	Moderate knee OA
All patients			
No. of patients	20	18	15
Age (year)^a^	34 ± 13	53 ± 13	59 ± 17
Female patients			
No. of patients	7 (35%)	6 (34%)	8 (53%)
Age (year)^a*^	34 ± 14	54 ± 14	62 ± 14
Male patients			
No. of patients	13 (65%)	12 (66%)	7 (47%)
Age (year)^a*^	32 ± 12	53 ± 14	54 ± 21

^a^Mean values ± standard deviation

*There were significant differences (*p* < 0.001) in age between the three subject groups

*OA*, osteoarthritis

### Reproducibility of KLG scoring

Inter-observer reproducibility for scoring the degree of radiographic knee OA according to KLG was good (weighted kappa, 0.78), while intra-observer reproducibility was excellent (weighted kappa, 0.85).

### qDESS T_2_ mapping and MOAKS in cartilage

**Fig. 3:**
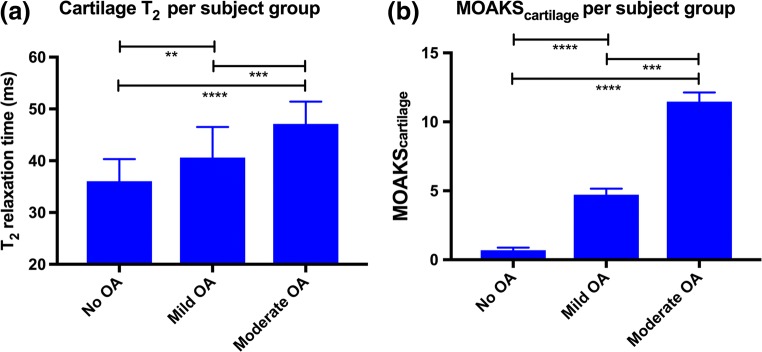
Discriminative power of quantitative and structural qDESS-based measurements in cartilage. Statistical significantly differences in (**a**) cartilage T_2_ and (**b**) MOAKS_cartilage_ scores were found among subject groups. Data is shown as overall mean values (pooled across all ROIs); vertical bars represent standard deviation. Horizontal bars represent statistically significance between two subject groups; **p < 0.01, ***p < 0.001, ****p < 0.0001. ms = milliseconds; OA = osteoarthritis; ROI = region of interest

**Fig. 4:**
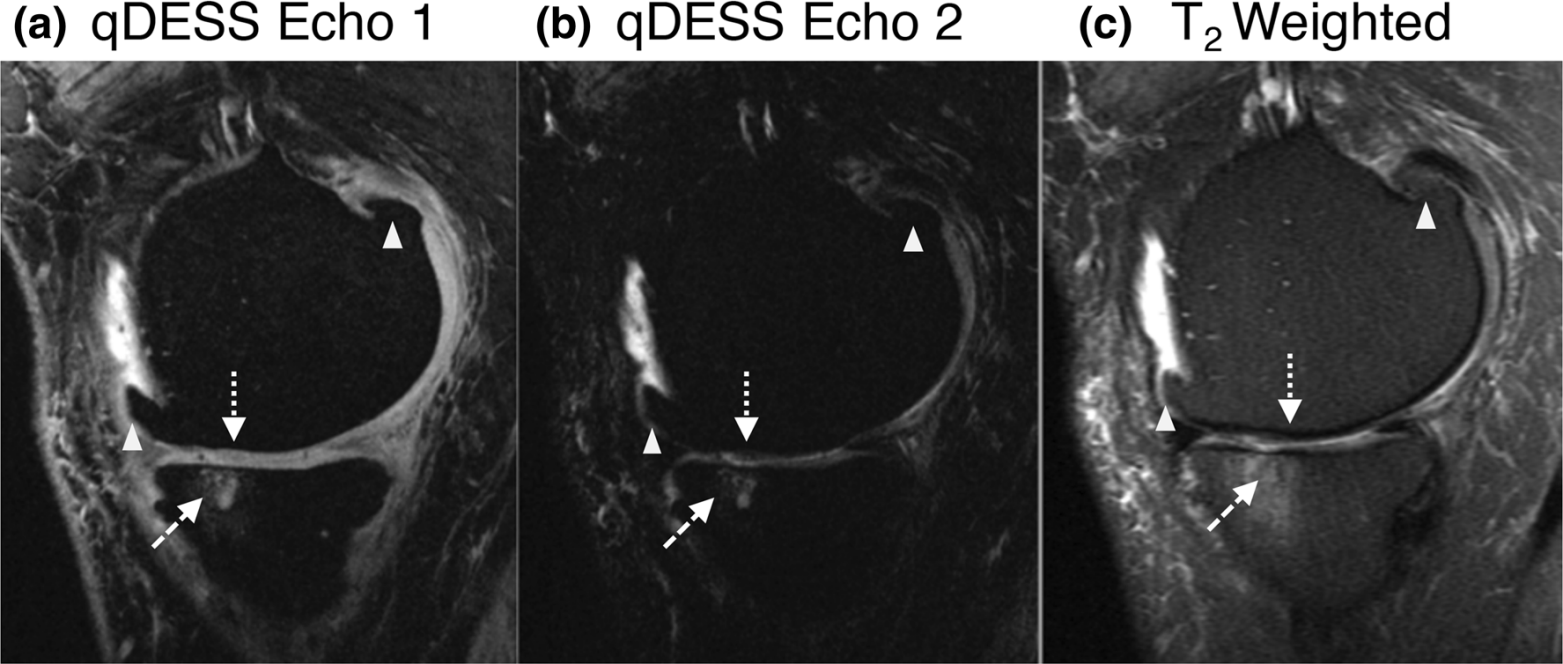
Example of MOAKS_cartilage_ assessment in a 71-year-old male with moderate OA on qDESS images (**a**, **b**), compared with corresponding fat-suppressed T_2_-weighted image (**c**) (TE 54 ms; flip angle 142°; FOV 14 cm; matrix 384 × 192). Sagittal images of first (**a**) and second (**b**) qDESS echo show thinning of medial femoral cartilage (dotted arrow). Subchondral cysts and surrounding BML (dashed arrow) and osteophytes (triangles) were not included in the analysis of the present study, but they were identified on qDESS images. Note the underestimation of BML size on qDESS images compared with T_2_-weighted image. OA = osteoarthritis; BML = bone marrow lesion

Overall qDESS cartilage (i.e., pooled across all ROIs) T_2_ values were 36.1 ± SD 4.3, 40.6 ± 5.9, and 47.1 ± 4.3 ms for no, mild, and moderate OA, respectively. The delta value (difference) in T_2_ was 4.6 ms between no OA and mild OA and 6.5 ms between mild OA and moderate OA. Overall qDESS cartilage T_2_ values were similar to T_2_ values in previous literature (33.8–38.8, 34.9–41.8, and 40.5–46.9 ms for no, mild, and moderate OA, respectively [7, 16, 28]). Differences in qDESS T_2_ values were statistically significant between the three subject groups (*p* < 0.01; Fig. 3a). Likewise, overall MOAKS_cartilage_ scores were consistently higher with increasing stages of OA with statistically significant differences found between the three subject groups (*p* < 0.001; Fig. 3b). The delta value (difference) in MOAKS_cartilage_ was 4 between no OA and mild OA and 6.8 between mild OA and moderate OA. A representative example of qDESS MOAKS_cartilage_ findings in a subject with moderate OA, compared with a corresponding fat-suppressed T_2_-weighted image, is provided in Fig. 4. Osteophytes were not included in the analyses of the present study, but they were identified on qDESS images. Subchondral cysts and surrounding bone marrow lesions (BMLs) were not included in the analyses of this study but identified as well (see Fig. 4). Overall qDESS T_2_ and MOAKS scores for cartilage, stratified by degree of OA, are summarized in Table 4.

**Table 4:** Cartilage T_2_ values and MOAKS_cartilage_ scores per ROI and overall scores, and their correlation with radiographic degree of OA

	No OA		Mild OA		Moderate OA		Correlated with radiographic OA^a^	
	T_2_^b^	MOAKS_cartilage_	T_2_^b^	MOAKS_cartilage_	T_2_^b^	MOAKS_cartilage_	T_2_^b^ vs. KLG	MOAKS vs. KLG
Cartilage ROI	Mean ± SD	Mean ± SD	Mean ± SD	Mean ± SD	Mean ± SD	Mean ± SD	Rho (95% CI)	Rho (95% CI)
Medial femur	36.5 ± 5.0	0.4 ± 0.7	43.4 ± 6.1	1.7 ± 1.5	50.6 ± 7.2	3.5 ± 2.9	0.71 (0.53–0.82)	0.62 (0.42–0.77)
Lateral femur	37.2 ± 4.4	0.3 ± 0.7	40.8 ± 5.4	1.3 ± 1.2	48.8 ± 8.4	2.4 ± 2.7	0.57 (0.35–0.73)	0.50 (0.26–0.69)
Medial tibia	34.7 ± 3.7	0.1 ± 0.2	39.4 ± 5.8	1.1 ± 2.4	44.2 ± 6.7	3.4 ± 3.7	0.53 (0.30–0.71)	0.51 (0.28–0.69)
Lateral tibia	35.8 ± 5.0	0.0 ± 0.0	38.8 ± 6.3	0.6 ± 1.2	48.8 ± 8.6	2.2 ± 2.8	0.43 (0.17–0.63)	0.51 (0.28–0.69)
Cartilage overall^c^	36.0 ± 4.3	0.7–0.2	40.6 ± 5.9	4.7 ± 0.4	47.1 ± 4.3	11.5 ± 0.7	0.75 (0.60–0.85)	0.82 ( 0.70–0.89)

^a^Data is shown as Spearman’s rho correlation coefficient between radiographic degree of OA (i.e., KLG) and corresponding T_2_ or MOAKS score, with 95% CI shown between brackets

^b^In milliseconds (ms)

^c^Pooled across all ROIs

*ROI*, region of interest; *KLG*, Kellgren-Lawrence grade; *OA*, osteoarthritis; *SD*, standard deviation; *95% CI*, 95% confidence interval

### T_2_ mapping and MOAKS in menisci

**Fig. 5:**
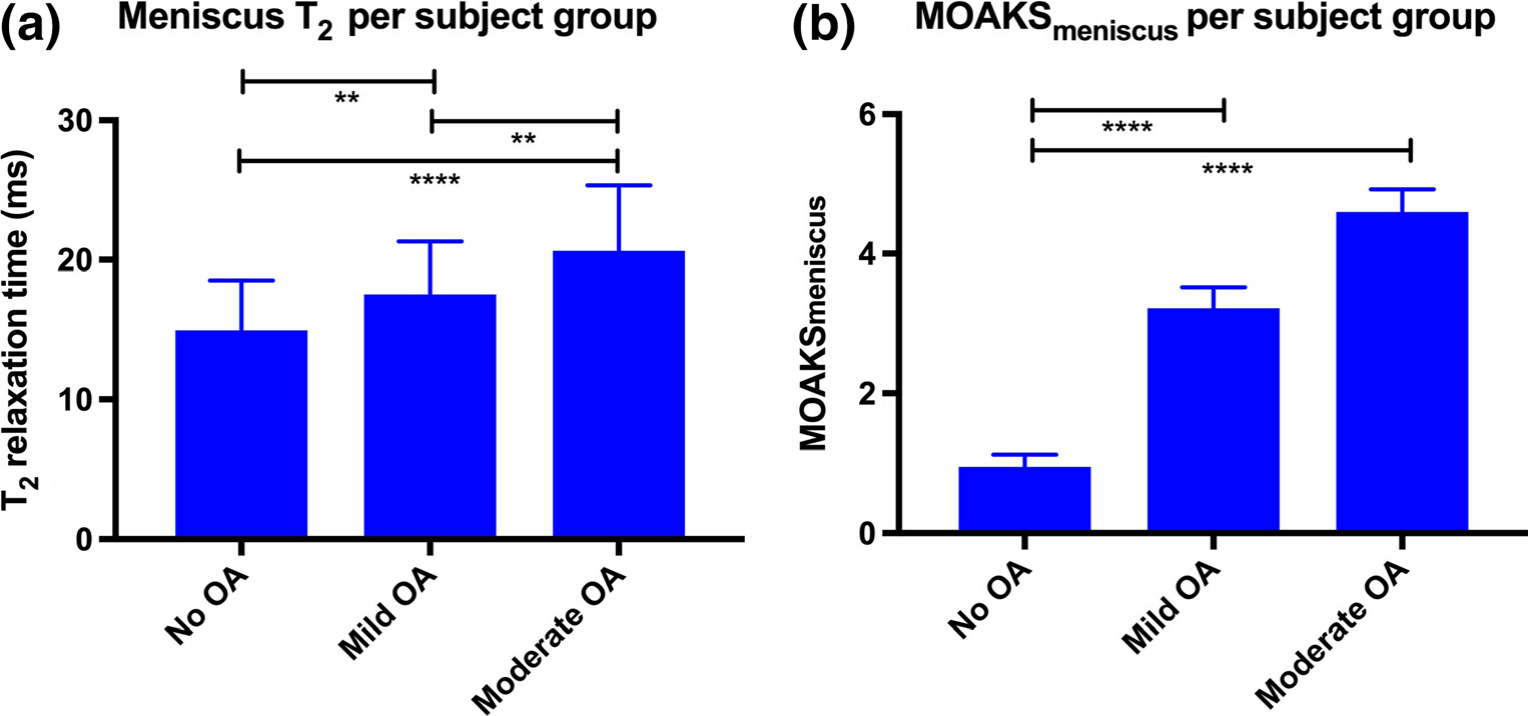
Discriminative power of quantitative and structural qDESS-based measurements in menisci. Statistical significantly differences in meniscus T_2_ (**a**) and MOAKS_meniscus_ (**b**) scores were found among subject groups. Data is shown as overall mean values (pooled across all ROIs); vertical bars represent standard deviation. Horizontal bars represent statistically significance between two subject groups; **p < 0.01, ***p < 0.001, ****p < 0.0001. ms = millisecond; OA = osteoarthritis; ROI = region of interest

In menisci, overall (i.e., pooled across all ROIs) qDESS T_2_ values were 15 ± SD 3.6, 17.5 ± 3.8, and 20.6 ± 4.7 ms for no, mild, and moderate OA, respectively. The delta value (difference) in T_2_ was 2.5 ms between no OA and mild OA and 3.1 ms between mild OA and moderate OA. Overall qDESS meniscus T_2_ values were similar to T_2_ values in previous studies (11.4–21.3, 13.5–22.4, and 16.8–24.2 ms for no, mild, and moderate OA, respectively [7, 16, 29]). Differences in qDESS T_2_ values were statistically significant between the three subject groups (*p* < 0.01; Fig. 5a). Differences in qDESS MOAKS_meniscus_ scores were statistically significant between the three subject groups (*p* < 0.001; Fig. 5b), except for the difference in MOAKS_meniscus_ scores between subjects with mild and moderate OA. The delta value (difference) in MOAKS_meniscus_ was 2.2 between no OA and mild OA and 1.5 between mild OA and moderate OA. An example of qDESS MOAKS_meniscus_ assessment in a subject with mild OA, compared with a corresponding proton-density-weighted image, is provided in Figure S1. Overall qDESS T_2_ values and MOAKS scores for menisci, stratified by degree of OA, are summarized in Table 5. With regard to meniscus extrusion, the presence of meniscus extrusion was consistent with the degree of OA. We found a medial extrusion of 0.3 ± SD 0.1, 0.9 ± 0.3, and 1.1 ± 0.3 in non-OA subjects, subjects with mild OA, and subjects with moderate OA, respectively. A lateral extrusion of 0.0 ± SD 0.0, 0.4 ± 0.2, and 0.7 ± 0.3 was found in non-OA subjects, subjects with mild OA, and subjects with moderate OA, respectively. Statistically significant differences in medial and lateral extrusion grade were found among the three subject groups (*p* = 0.04 and *p* = 0.03 for medial and lateral extrusion, respectively).

**Table 5:** Meniscus T_2_ values and MOAKS_meniscus_ scores per ROI and overall scores, and their correlation with radiographic degree of OA

	No OA		Mild OA		Moderate OA		Correlated with radiographic OA^a^	
	T_2_^b^	MOAKS_meniscus_	T_2_^b^	MOAKS_meniscus_	T_2_^b^	MOAKS_meniscus_	T_2_^b^ vs. KLG	MOAKS vs. KLG
Meniscus ROI	Mean ± SD	Mean ± SD	Mean ± SD	Mean ± SD	Mean ± SD	Mean ± SD	Rho (95% CI)	Rho (95% CI)
Medial anterior	14.2 ± 2.4	0.1 ± 0.4	16.1 ± 2.5	0.4 ± 1.1	17.7 ± 5.2	0.7 ± 1.2	0.39 (0.13–0.60)	0.29 (0.02–0.53)
Medial posterior	16.3 ± 5.9	0.5 ± 0.9	19.6 ± 4.8	1.1 ± 1.2	22.9 ± 7.6	1.3 ± 1.2	0.50 (0.25–0.68)	0.34 (0.07–0.57)
Lateral anterior	14.8 ± 3.5	0.2 ± 0.7	17.2 ± 3.8	1.1 ± 1.0	20.6 ± 5.3	1.3 ± 1.5	0.51 (0.27–0.69)	0.45 (0.19–0.65)
Lateral posterior	14.6 ± 2.4	0.2 ± 0.7	17.2 ± 4.0	0.7 ± 1.0	21.2 ± 7.9	1.3 ± 1.0	0.48 (0.23–0.67)	0.52 (0.29–0.70)
Meniscus overall^c^	15.0 ± 3.6	1.0 ± 1.2	17.5 ± 3.8	3.2 ± 0.3	20.6 ± 4.7	4.6 ± 0.3	0.64 (0.44–0.78)	0.65 ( 0.45–0.79)

^a^Data is shown as Spearman’s rho correlation coefficient between radiographic degree of OA (i.e., KLG) and corresponding T_2_ or MOAKS score, with 95% CI shown between brackets

^b^In milliseconds (ms)

^c^Pooled across all ROIs

*ROI*, region of interest; *KLG*, Kellgren-Lawrence grade; *OA*, osteoarthritis; *SD*, standard deviation; *95% CI*, 95% confidence interval

### qDESS T_2_ mapping and MOAKS in cartilage and meniscus ROIs

qDESS T_2_ values and MOAKS scores for each cartilage and meniscus ROI, stratified by degree of OA, are summarized in Tables 4 and 5, respectively. In all cartilage and meniscus ROIs, statistically significant correlations were found between qDESS T_2_ values and radiographic OA and between MOAKS scores and radiographic OA. The strongest correlation (*r* = 0.71) between MRI findings and radiographic OA was found in the medial femoral cartilage; the weakest correlation (*r* = 0.29) was found in the anterior horn of the medial meniscus.

## Discussion

In the present study, we demonstrated that quantitative and structural measurements in cartilage and meniscus, obtained with a single 5-min qDESS sequence, can differentiate between OA stages. T_2_ values in cartilage and menisci were similar to T_2_ values reported in previous studies [5–8].

The disease distribution of OA within the knee joint is often compartmental, with high variability regarding compartmental involvement [6, 20, 21]. Therefore, we assessed the validity of qDESS-based biomarkers in various cartilage and meniscus ROIs. The discriminative power to distinguish degree of OA was the greatest in the medial femoral cartilage, and the least in the anterior horn of the medial meniscus. These findings were most likely caused by the uneven distribution of OA features; the anterior horn of the medial meniscus showed relatively low T_2_ values and MOAKS scores in subjects with mild or moderate OA while the medial femoral cartilage showed relatively high T_2_ values and MOAKS scores in those subjects. Despite the differences in discriminative power, T_2_ values and MOAKS outcomes in all ROIs were found to be statistical significantly correlated with radiographic knee OA.

The qDESS sequence in the present study was optimized to simultaneously generate high-resolution images and quantitative measurements, by combining high spatial resolution with high SNR, in one single, rapid scan. While twice as fast, the resolution and voxel volume of this qDESS sequence (0.18 μL) was over 10x better than the resolution of established quantitative T_2_ sequences [7, 30]. In a previous study, qDESS has shown high T_2_ accuracy compared with multi-echo spin echo sequences, as well as high accuracy for MOAKS measurements compared with conventional spin echo–based sequences, with high intra- and inter-observer reproducibility [16, 25]. qDESS has been thought to underestimate the size of bone marrow lesions (BMLs), which seems to be the case in our study as well (see Fig. 4, not studied), likely due to T_2_* susceptibility effects [15]. A separation of the two qDESS echoes may enhance accuracy of BML detection compared with previous qDESS studies [31]. Although outside the scope of this study, further work is needed to test and optimize BML detection with qDESS.

Building upon the work of Chaudhari et al [16], the present study assesses the discriminative power of a 5-min qDESS sequence to obtain T_2_ values and MOAKS in a clinical knee OA population. We validated T_2_ measurements and MOAKS against radiographic OA, which remains the gold imaging standard for diagnosing and monitoring knee OA [18, 19]. In OA research, KLG2 is considered the cut-off point for the presence of radiographic knee OA [4, 18, 19, 32]. Although potentially a relevant group in the context of early OA imaging, we did not include patients with KLG1, indicating doubtful radiographic OA. The reproducibility of scoring KLG1 (i.e., doubtful narrowing of joint space and possible osteophytic lipping) is relatively poor, most likely due to differences in the interpretation of radiographic findings, especially concerning osteophytic lipping [18]. Also, patients with severe radiographic OA (i.e., KLG4) were not included in the present study, as bony deformity and bone-to-bone contact precludes accurate segmentation of cartilage.

OA is among the top ten burdensome diseases, with the knee being the most affected joint [1]. In the light of increased numbers associated MR imaging studies [2, 33], reducing MR imaging acquisition time is highly relevant. Reducing scan time saves costs and increases patient comfort and may reduce motion artifacts in longer acquisitions [16]. Because qDESS rapidly provides rich structural and quantitative information, there is a great promise for using this technique in large clinical OA studies. Recent advances in deep learning and simultaneously imaging both knees with qDESS may further reduce scan time, without loss of image quality or quantitative accuracy [34–36].

This study has some limitations that must be acknowledged. First, segmentation of quantitative analysis and MOAKS scoring was performed by a single, experienced researcher. As evidence of high intra- and inter-observer reproducibility for cartilage and meniscus segmentation and MOAKS assessment with qDESS images has been reported previously [16], analyses performed by a single researcher were considered sufficient. Second, our validation study was cross-sectional. The lack of a longitudinal aspect may limit interpretation regarding the potential use of qDESS in clinical trials. Therefore, future studies on the sensitivity of qDESS-based biomarkers for longitudinal changes in the knee are required. Third, KLG was used as reference standard, which is considered the gold standard for imaging-based knee OA classification [4]. Radiographically detected joint space narrowing (JSN) is currently the only structural endpoint accepted by the European and US regulatory bodies (European Medicines Agency and FDA) to assess knee OA progression [37] and is commonly used in qMRI validation studies [6, 7]. We opted for this method because we aimed to explicitly use qDESS in a clinically relevant matter. However, an important drawback of the KLG method is the low reproducibility of JSN measures reported in literature, in particular in longitudinal assessment of knee OA [4, 38]. Given the cross-sectional design of our study without longitudinal measures, challenges concerning longitudinal KLG measures are unlikely. To optimize reproducibility, we used standardized radiographs (weight-bearing AP). To assess reproducibility, both inter- and intra-observer reproducibility of KLG were carefully evaluated in the present study (weighted kappa of 0.78 and 0.85 for inter- and intra-observer reproducibility, respectively). Finally, although osteophytes and BMLs are important OA features, they were not studied. The primary objective of this study was to assess the validity of qDESS for cartilage and menisci in OA subjects. We focused on those tissues as they have conclusively been shown to be strong indicators for OA and because of their possibilities in both quantitative (T_2_) and semi-quantitative (MOAKS) [4, 7, 8, 11, 39]. To assess the external validity of our study results, further studies evaluating other relevant OA features will be essential, in particular regarding BML detection. In addition, future validation studies on qDESS T_2_ values in OA patients against histological degree of degeneration (the gold standard for tissue changes) are desirable.

In conclusion, quantitative T_2_ and structural assessment of cartilage and meniscus with a single 5-min qDESS scan can distinguish between different grades of OA and show significant correlations with the reference standard. These results demonstrate the potential of qDESS as an efficient and accurate imaging tool for OA research.

## Electronic supplementary material

ESM 1 (DOCX 753 kb)

## Abbreviations

ACR: American College of Rheumatology
KLG: Kellgren and Lawrence grade
MOAKS: MRI Osteoarthritis Knee Score
MRI: Magnetic resonance imaging
OA: Osteoarthritis
qDESS: Quantitative double-echo steady-state
ROI: Region of interest
SD: Standard deviation
TE: Echo time
95% CI: 95% confidence interval
