# Supplementary material for: Time-saving opportunities in knee osteoarthritis: T2 mapping and structural imaging of the knee using a single 5-min MRI scan
Source: Eur Radiol. 2019 Dec 16;30(4):2231–40. doi: 10.1007/s00330-019-06542-9 (PMC7062657; doi:10.1007/s00330-019-06542-9)
Supplement: Supplementary file 1 — (DOCX 753 kb) [file 330_2019_6542_MOESM1_ESM.docx]

**Supplementary material 1**: **MOAKS grading for cartilage**

For structural, semi-quantitative assessment of cartilage in the present study, cartilage subscores (MOAKS_cartilage_), directly derived from the MOAKS total scores, were used (described in Table S1). MOAKS_cartilage_ includes the size of cartilage lesions and the percentage of cartilage lesions being full thickness [1].


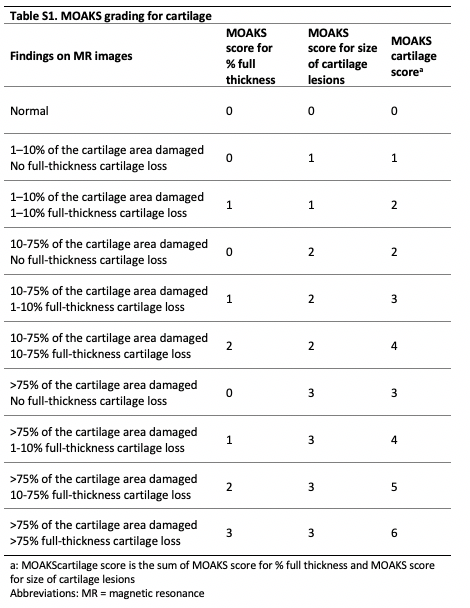


**Supplementary material 2:** **MOAKS grading for meniscus**

For structural, semi-quantitative assessment of the meniscus in the present study, meniscus subscores (MOAKS_meniscus_) based on MOAKS total scores were used, including meniscus signal, tears, and (partial) maceration (described in Table S2). The rationale behind MOAKS_meniscus_ criteria and the hierarchy in MOAKS_meniscus_ scoring used in the present study was based on the clinical important effects these meniscus findings have as described in literature [2; 3].


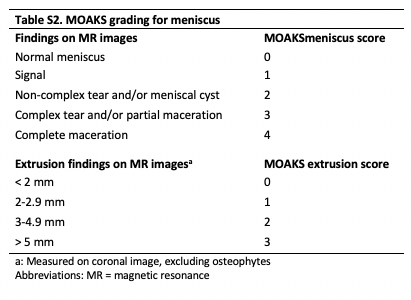


**References**

1 Hunter DJ, Guermazi A, Lo GH et al (2011) Evolution of semi-quantitative whole joint assessment of knee OA: MOAKS (MRI Osteoarthritis Knee Score). Osteoarthritis and Cartilage 19:990-1002

2 Antony B, Driban JB, Price LL et al (2017) The relationship between meniscal pathology and osteoarthritis depends on the type of meniscal damage visible on magnetic resonance images: data from the Osteoarthritis Initiative. Osteoarthritis and Cartilage 25:76-84

3 Habata T, Uematsu K, Hattori K, Takakura Y, Fujisawa Y (2004) Clinical features of the posterior horn tear in the medial meniscus. Archives of Orthopaedic and Trauma Surgery 124:642-645


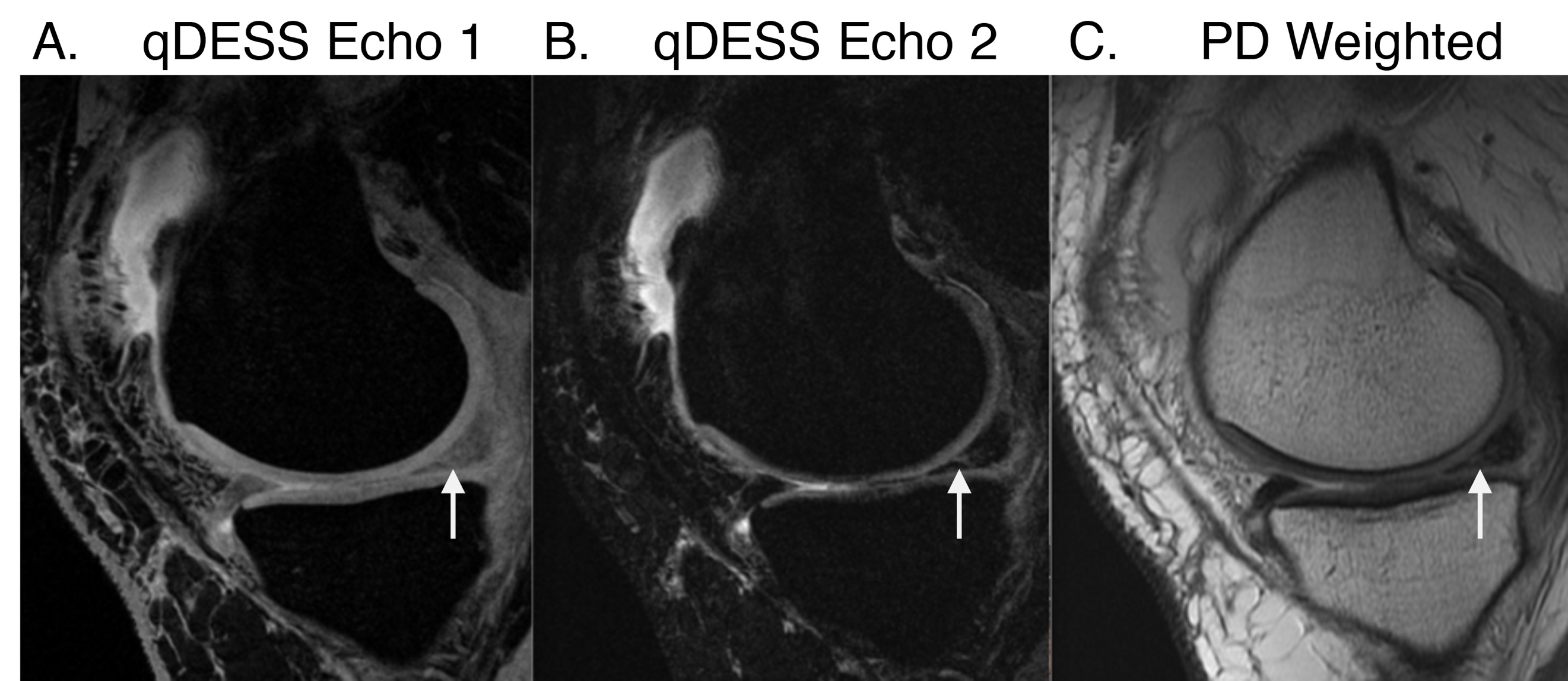


***Figure S1.*** Example of MOAKS_meniscus_ findings in 47-year-old male with mild OA, on sagittal images of (a) first and (b) second qDESS echo, compared to (c) corresponding proton density-weighted image (TE 35 ms; flip angle 142°; FOV 14 cm; matrix 384x224), showing a complex tear (solid arrow) in the posterior horn of the medial meniscus. MOAKS_meniscus_ scoring in the present study included meniscus signal, tears, and (partial) maceration. The second qDESS echo especially was useful in identifying meniscus pathology. Abbreviations: OA = osteoarthritis; PD = proton density.
